# Visual Function and Patient Satisfaction with Multifocal Intraocular Lenses in Patients with Glaucoma and Dry Age-Related Macular Degeneration

**DOI:** 10.1155/2021/9935983

**Published:** 2021-06-10

**Authors:** Carmen Sánchez-Sánchez, Laureano A. Rementería-Capelo, Beatriz Puerto, Cristina López-Caballero, Aida Morán, José María Sánchez-Pina, Inés Contreras

**Affiliations:** ^1^Clínica Rementería, Madrid, Spain; ^2^Hospital Universitario Ramón y Cajal, Madrid, Spain; ^3^Instituto Ramón y Cajal de Investigaciones Sanitarias (IRYCIS), Madrid, Spain

## Abstract

**Purpose:**

To report visual function and self-reported satisfaction of patients with glaucoma and dry age-related macular degeneration (dAMD) implanted with multifocal intraocular lenses (MIOL).

**Methods:**

Patients with glaucoma or dAMD as well as healthy individuals implanted with MIOL were invited to participate. Explorations performed were uncorrected and corrected distance visual acuity (UDVA and CDVA), low-contrast visual acuity (LCVA), binocular contrast sensitivity, and defocus curves. Patients completed the Catquest-9 questionnaire and reported on the presence of dysphotopsias and the need for spectacles.

**Results:**

38 subjects were included: 11 in the healthy/control group and 9 each in the preperimetric glaucoma, perimetric glaucoma, and dAMD groups. Controls had statistically better monocular UDVA, CDVA, and LCVA than patients with glaucoma and dAMD, as well as better binocular acuity in the defocus curves between −2.00 D and +0.50 D. Differences between controls and patients with preperimetric glaucoma were not statistically significant. Between −3.0 D and +0.5 D, all groups except dAMD achieved acuities better than 0.2 logMAR. Patients with dAMD had worse contrast sensitivity than all others for 3 cycles per degree (cpd), and patients with glaucoma had worse values than all others for 12 cpd; other differences did not reach statistical significance. Healthy subjects and patients with preperimetric glaucoma perceived halos more often than patients with glaucoma or dAMD, while suffering less from glare. Patients with glaucoma and dAMD found more difficulties when driving at night and required spectacles for near more often than the other subjects. Patients with dAMD were less satisfied with their vision.

**Conclusions:**

MIOLs may be implanted in patients with preperimetric glaucoma with little fear of patient dissatisfaction. In glaucoma and dAMD, MIOLs might be considered with caution, after explaining the increased risk of glare and the higher need for spectacle correction for reading.

## 1. Introduction

The search for spectacle independence after cataract surgery is driving the development of multiple intraocular lens (IOL) designs, such as trifocal, extended depth of focus, or advanced monofocal. Each lens has advantages and disadvantages, and its characteristics must be carefully discussed with each patient, taking into account the possible presence of ocular pathology other than cataracts [1]. Diffractive designs have three main drawbacks: they split light, leading to reduced contrast sensitivity; there may be a reduction in quality of vision, leading to problems with overall quality or with dim lighting, and patients often report dysphotopsias [2]. These issues are especially relevant in patients with glaucoma or retinal pathologies, and usually, ophthalmologists do not consider the implantation of multifocal diffractive designs in these patients [3].

Several meta-analyses have established that multifocal IOLs lead to lower contrast sensitivity [4, 5], but there have been other reports with different results. Ouchi and Kinoshita reported on the implantation of the refractive M-plus lens in 15 eyes with coexisting ocular pathologies, including glaucoma, branch retinal vein occlusion, and fundus albipunctatus [6]. Contrast sensitivity in all patients was comparable to levels described for healthy subjects, and no patient reported poor visual quality. Kamath et al. reported that the Array multifocal IOL produced similar distance visual outcomes as a monofocal IOL in patients with cataract and diseases such as macular degeneration, glaucoma, and diabetic retinopathy [7]. Gayton et al. also reported favorable visual outcomes with multifocal IOLs in patients with age-related macular degeneration (AMD) using a −2.00 diopters (D) refractive target as a magnification strategy [8].

The purpose of our study was to report visual function, including contrast sensitivity and low-contrast visual acuity, in subjects with glaucoma or dry AMD, comparing them with a group of healthy subjects.

## 2. Methods

Patients who were seen in our clinic between 2018 and 2020 who had been implanted with a multifocal IOL and who also suffered from glaucoma or dry AMD were invited to participate in our study. Healthy individuals who came in for routine follow-up after cataract surgery during this period were also asked to participate. All subjects signed an informed consent form before inclusion. The study was approved by the Ethics Committee of the Hospital Clínico San Carlos, Madrid, and followed the tenets of the Declaration of Helsinki.

All subjects included in the study had undergone cataract surgery at least 6 months prior to inclusion. Patients with glaucoma were classified into two groups, according to the results of the visual fields performed at the same visit as the study procedures. Patients with increased optic disc cupping with retinal nerve fiber layer loss on optical coherence tomography (OCT) but no visual field defects were included in the preperimetric glaucoma group. Patients with both optic nerve and visual field damage composed the glaucoma group. Glaucomatous field damage was classified according to the Hodapp classification. Patients with dry AMD were included in the AMD group; patients with choroidal neovascularization (present or past) were excluded from the study.

Initially, uncorrected distance visual acuity with an ETDRS chart was measured, followed by subjective refraction (best distance correction). Contrast visual acuity evaluation was performed with a Freiburg Acuity Test software package [9, 10] run on a laptop screen calibrated to be presented at 4 meters. A black Landolt C was presented to the subjects, and they indicated its orientation on a numeric keypad. This test minimizes the observer's bias because the presented optotype depends on the patient's previous responses; when the observer responds to a certain optotype, the software automatically modifies the size of the next one according to parameter estimation by a sequential test method. Visual acuity was evaluated with this system at low (10%) and medium (50%) contrast, mono- and binocularly.

Binocular contrast sensitivity function was measured for spatial frequencies of 3, 6, 12, and 18 cycles per degree (cpd) using the functional acuity contrast test (Test SV-1000) of the CC-100 HW 5.0 Series system. Contrast sensitivity values were converted to Absolute log10 (log10 CS) for analysis.

Binocular defocus curves were performed starting at −5.00 D in 0.50 D steps until +3.00 D defocus. Patients were also asked to complete the Catquest-9 questionnaire and were asked about the presence of dysphotopsias and the need for spectacle correction.

SPSS version 26 (IBM, Chicago, USA) was used for statistical analyses. Due to the small number of patients included, nonparametric tests were used. Differences between groups were evaluated with the Kruskal–Wallis test. *P* values were adjusted automatically by the software with the Bonferroni correction for multiple comparisons. A corrected *p* value <0.05 was considered statistically significant.

## 3. Results

The study included a total of 38 subjects: 11 in the healthy control group, 9 patients with preperimetric glaucoma, 9 patients with glaucoma, and 9 patients with dry AMD.  Table 1 provides the age, gender, and type of lens implanted for each group. Patients in the healthy control group were younger than patients with glaucoma and dry AMD. The intraocular lenses implanted included AcrySof ReSTOR +3.00 (bifocal); AcrySof Panoptix (trifocal), Physiol FineVision (trifocal), and Technis Symfony (extended depth of focus), all of which depend on diffractive technology.

**Figure 1:**
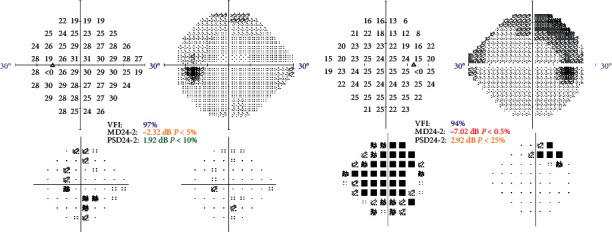
Visual fields of one of the patients included, with moderate glaucoma, a 78-year-old woman.

**Figure 2:**
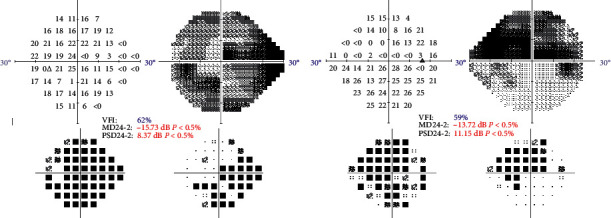
Visual field defects of one of the patients included, with advanced glaucoma, an 86-year-old man.

Of the 9 patients with dry AMD included, one patient had early AMD in both eyes, 2 patients early AMD in one eye and intermediate AMD in the other eye, five patients had intermediate AMD in both eyes, and one patient had advanced AMD in both eyes. Of the patients with glaucoma, 4 patients had early glaucomatous visual field damage in both eyes, one patient had an early defect in one eye and a moderate defect in the other eye, two patients had an early defect in one eye and an advanced defect in the other, and two patients had advanced defects in both eyes. Figures 1 and 2 show two examples of visual field defects.

**Figure 3:**
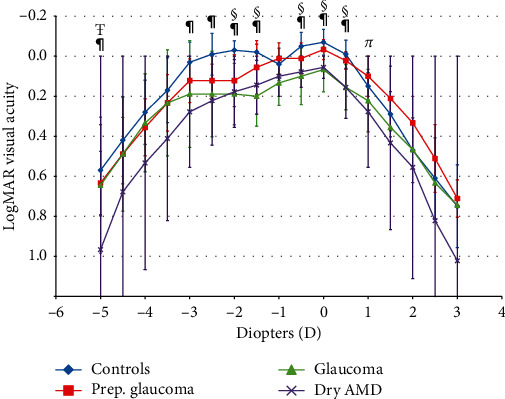
Defocus curves for all groups.

Visual acuity values are given in Table 2. Healthy subjects had better visual acuities than patients with glaucoma and dry AMD for all values except for binocular 10% contrast-corrected visual acuity. Patients in the preperimetric glaucoma group had worse visual acuities than healthy subjects and better acuities than the glaucoma and dry AMD groups, although differences did not reach statistical significance.  Figure 3 shows the binocular defocus curves for all groups. Healthy subjects had statistically significant better values than patients with dry AMD for most defocus values between −3.00 D and +0.50 D. Healthy subjects also had better values than patients with glaucoma for most defocus values between −2.0 D and +0.50 D defocus. However, healthy subjects did not have statistically better binocular visual acuity than patients with preperimetric glaucoma. Patients with preperimetric glaucoma tended to have better visual acuity for all distances than patients with glaucoma and dry AMD, but the differences were not statistically significant.

**Figure 4:**
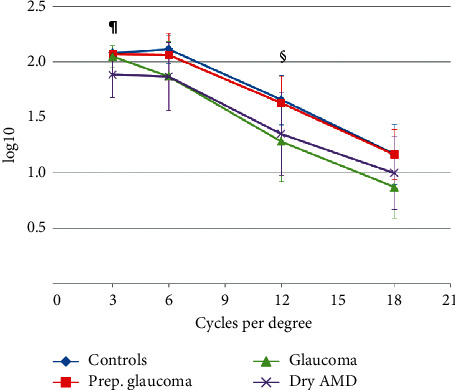
Binocular contrast sensitivity.

Figure 4 and Table 3 show binocular contrast sensitivity values. Patients with dry AMD had worse values than all other groups for 3 cycles per degree (cpd) and patients with glaucoma had worse values than all other groups for 12 cpd; other differences did not reach statistical significance.

**Figure 5:**
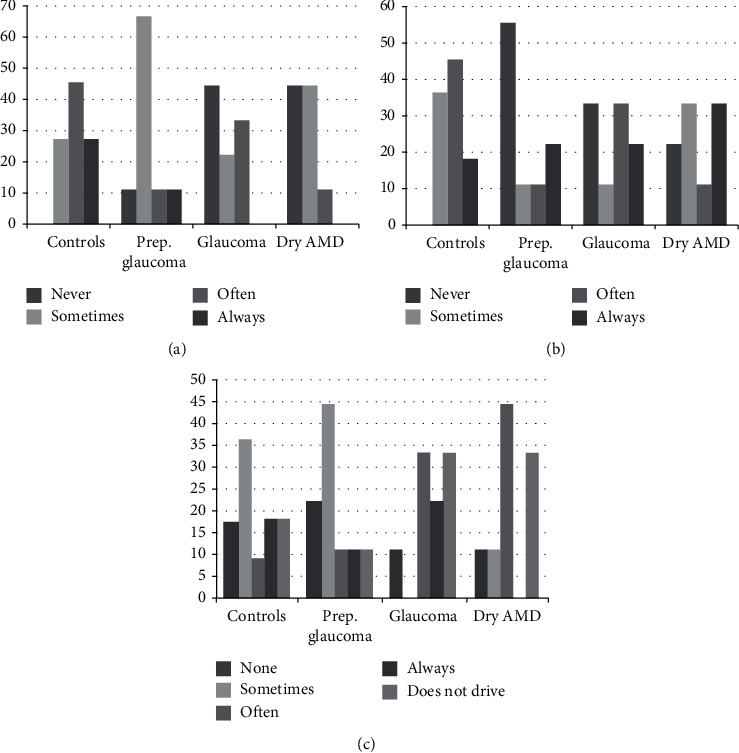
Self-reported presence of halos and glare and difficulty for driving at night. (a) Halo. (b) Glare. (c) Difficulty for driving at night.

**Figure 6:**
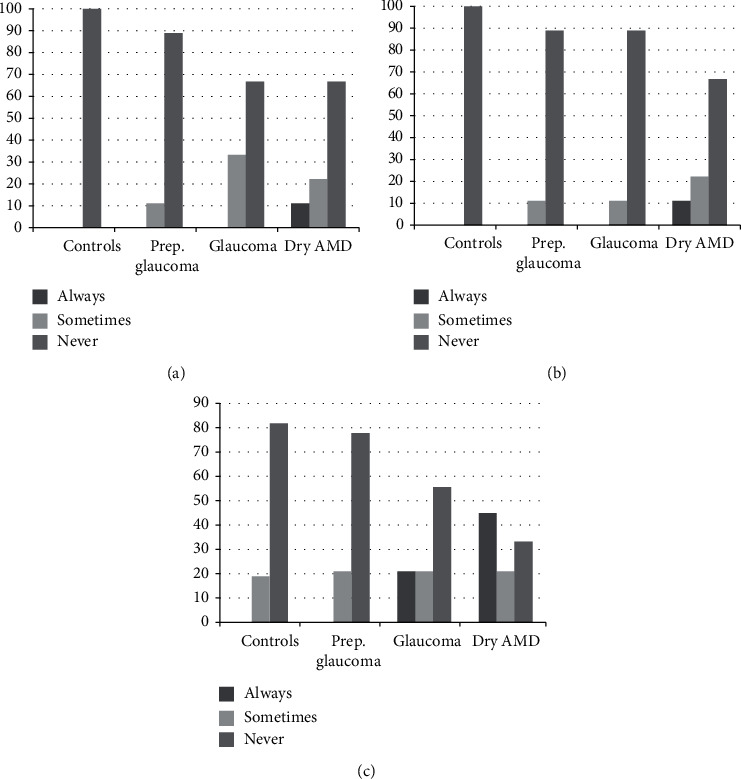
Need for spectacle correction for different distances. (a) Distance. (b) Intermediate. (c) Near.

As regards patient-reported outcomes, Figure 5 shows the incidence of halos, glare, and difficulty for driving at night. Healthy subjects and patients with preperimetric glaucoma perceived halos more often than patients with glaucoma or dry AMD, while suffering less from glare. Patients with glaucoma and dry AMD found more difficulties when driving at night or did not drive at all more often than healthy subjects and patients with preperimetric glaucoma.  Figure 6 shows the need for spectacle correction. Patients with glaucoma and dry AMD required spectacle correction for near more often than the other groups. The results of the Catquest-9 questionnaire are given in Table 4. Patients with dry AMD were less satisfied with their vision than healthy subjects and patients with preperimetric glaucoma, but there were no other statistically significant differences between groups.

## 4. Discussion

There is a lack of scientific evidence in the form of large trials on the impact of multifocal IOLs in patients with ocular disease such as glaucoma or retinal diseases. In a recent review, Grzybowski et al. were unable to find evidence suggesting that patients with macular diseases should be advised against multifocal IOLs and concluded that more research is needed especially to address the effect of multifocal IOLs on contrast sensitivity and visual function [11].

The purpose of our study was to report on visual function after multifocal IOL implantation in patients with glaucoma and dry AMD and to compare them with healthy subjects. As regards monocular distance visual acuity, healthy subjects had statistically significant better values than patients with glaucoma and dry AMD, both for uncorrected and corrected acuity. This was to be expected, but there are several interesting facts. There were no statistically significant differences between healthy subjects and patients with preperimetric glaucoma. For low-contrast visual acuity, differences between the healthy control group and the glaucoma and dry AMD groups dropped by half when assessed binocularly. This effect of binocular function can also be seen in the defocus curves, where differences between groups are lower. Between −3.0 D and +0.5 D, all groups except dry AMD patients achieved a visual acuity better than 0.2 logMAR.

Comparing the visual acuity results of the patients with ocular pathologies in our study with other studies with multifocal IOL in healthy subjects is difficult since most patients included in these studies are younger, as our control group. Thus, in Jonker et al.'s study, the mean age of patients included in the ReSTOR group was 64.0 ± 8.8 years [12]. Mean uncorrected monocular distance visual acuity was 0.08 ± 0.11, which is slightly worse than in our control group but better than in patients with glaucoma or dry AMD. In Pepose et al.'s study, the mean age of patients included in the ReSTOR group was 64.2 ± 7.0 years; mean uncorrected monocular distance visual acuity was 0.016 ± 0.121, similar to our control group and better than in patients with ocular disease [13].

As regards contrast sensitivity, although several meta-analyses have established that multifocal IOLs lead to reduced contrast sensitivity compared to monofocal IOLs [4, 5], other studies present conflicting results [11]. Comparing contrast sensitivity outcomes is difficult due to the differences in the tests used and different illumination levels. It seems that contrast sensitivity with multifocal IOLs is lower than with monofocal IOLs in at least some conditions.

In our study, we found that contrast sensitivity was decreased for 3 cpd in patients with dry AMD and for 12 cpd in patients with glaucoma. However, it must be taken into account that contrast sensitivity decreases with age at all spatial frequencies, with a greater drop at higher spatial frequencies, and our healthy subjects were younger than patients with AMD and glaucoma. The lower value we found for the glaucoma group for 12 cpd would be in accordance with the lower contrast sensitivity values at high spatial frequencies reported for glaucomatous eyes [14].

Once again, comparison with other studies in healthy subjects with multifocal IOLs is difficult due to the age difference. Curiously, the values for both healthy subjects and patients with ocular disease in our study were better than those reported by Jonker et al. and Pepose et al. with a similar test [12, 13].

As mentioned before, Ouchi and Kinoshita reported the results of the implantation of a sectorial refractive multifocal IOL in 15 eyes of 11 patients with cataract and other ocular pathologies which might affect visual function [6]. The outcome results are pooled, so that it is difficult to ascertain the impact of each individual disease. They found contrast sensitivity dropped below normal levels for 18 cpd; however, their patients were far younger than those included in our study (mean 51.4 ± 11.84 years). The mean uncorrected and corrected distance visual acuities were −0.001 ± 0.11 and −0.07 ± 0.11, respectively, at 6 months postoperatively. These values are far better than those achieved by all but the healthy subjects included in our study, probably because Ouchi et al. included 5 patients with high myopia and 2 patients with keratoconus who might not have relevant damage to foveal function [6].

Gayton et al. implanted the AcrySof ReSTOR (an apodized, diffractive multifocal IOL with *a* + 3 addition power) in 20 eyes of 13 patients with AMD, targeting a spherical equivalent of −2.0 D in order to provide better near vision. This strategy improved or maintained near vision without severely compromising distance vision; however, contrast sensitivity was not evaluated [8].

Patel et al. reported on the use of multifocal IOLs in combined phacovitrectomy in patients with cataracts and epiretinal membranes [15]. Six eyes of five patients implanted with the AcrySof ReSTOR were included in their retrospective report. Postoperatively, uncorrected distance visual acuity improved to 0.038 at 6 months. All patients achieved J2 or better uncorrected near visual acuity (equivalent to 0.2 logMAR). Three patients experienced dysphotopsias, including glare, halos, or streaks of light.

In our study, we also evaluated the need for spectacle correction, the presence of halos and glare, and subjective evaluation of difficulties for daily living as assessed by the Catquest-9 questionnaire. We found that the healthy and preperimetric glaucoma groups reported more halos, while patients with glaucoma and dry AMD reported more glare. This might be due to increased perception in relatively healthy eyes of the effect on light dispersion of diffractive technology. On the other hand, damaged photoreceptors might be more susceptible to glare. Patients with glaucoma and AMD also found it more difficult to drive at night; this might be due not only to the effect of multifocality but also to visual field defects and decreased visual acuity and contrast visual acuity in the setting of their disease.

As regards satisfaction, only dry AMD patients reported a lower level than the other groups, while they reported similar difficulties for the activities of daily life. This corresponds to the results of the defocus curves and contrast sensitivity; although values are decreased compared to healthy subjects, they are for the most part within limits for correct function. The higher need of AMD patients for spectacle correction for near vision also reflects the results of the binocular defocus curve.

The main limitation of our study is the low number of patients included in each group. This is due to the fact that in our practice, we indicate multifocal IOLs with caution in patients with retinal or optic nerve disease. Most patients included developed their disease after multifocal IOL implantation. However, we believe the results show that multifocal IOLs may be implanted in patients with preperimetric glaucoma with little fear of patient dissatisfaction. In patients with glaucoma and dry AMD, they might be considered with caution in subjects with early disease, after warning them of the increased risk of glare and the need for spectacle correction for reading. Ophthalmologists must decide on the best lens according to the patient's motivation and the possibility and rate of disease progression.

## 5. Conclusions

Patients with glaucoma or dry AMD implanted with a multifocal IOL had lower monocular visual acuity than healthy controls and lower contrast sensitivity values in certain conditions. Although patient satisfaction was lower in dry AMD patients, overall binocular function seemed to provide adequate performance, since self-reported difficulties for daily activities were similar to those reported by healthy subjects. There were no clinically significant differences in visual performance between patients with preperimetric glaucoma and healthy controls. Therefore, multifocal IOL may be considered for patients with preperimetric glaucoma with little fear of patient dissatisfaction and may also be considered in selected cases of glaucoma and dry AMD.

## Figures and Tables

**Table 1:** Age, gender, and lens distribution.

	Healthy	Preperimetric glaucoma	Glaucoma	Dry AMD	*P*
Number of patients	11	9	9	9	NA
Age (years)	67.64 (6.36)	68.33 (8.37)	76.33 (6.61)	75.33 (6.91)	**0.027** ^**1**^
	59–78	57–79	68–88	62–82	

Gender					
Female	54.5%	44.4%	66.7%	44.4%	0.70
Male	45.5%	55.6%	33.3%	55.6%	

Type of lens					
Bifocal	27.3%	77.8%	77.8%	33.3%	0.072
Trifocal	72.7%	22.2%	22.2%	55.6%	
EDOF	0%	0%	0%	11.1%	

^1^ *P* = 0.022 for controls vs. glaucoma and *P* = 0.019 for controls vs. dry AMD. AMD, age-related macular degeneration; EDOF, extended depth of focus.

**Table 2:** Visual acuity LogMAR values.

	Healthy	Preperimetric glaucoma	Glaucoma	Dry AMD	*P*
Monocular UDVA	0.00 (0.09)	0.13 (0.21)	0.32 (0.35)	0.22 (0.23)	**<0.001** ^¶^
	0.30 to −0.10	0.80 to 0.00	1.30 to 0.00	0.80 to 0.00	

Monocular CDVA	−0.03 (0.04)	0.02 (0.05)	0.18 (0.25)	0.18 (0.23)	**<0.001** ^¶^
	0.00 to −0.10	0.20 to 0.00	0.80 to 0.00	0.80 to 0.00	

Spherical equivalent	−0.04 (0.23)	−0.25 (0.57)	0.23 (0.78)	−0.05 (0.13)	0.09
	−0.75 to 0.50	−2.00 to 0.50	−1.00 to +2.50	−0.50 to 0	

Monocular C50 CVA	0.05 (0.08)	0.12 (0.15)	0.32 (0.30)	0.37 (0.45)	**<0.001** ^¶^
	0.20 to −0.10	0.50 to 0.00	1.00 to 0.00	1.30 to 0.00	

Monocular C10 CVA	0.20 (0.09)	0.24 (0.17)	0.47 (0.39)	0.53 (0.41)	**0.002** ^¶^
	0.40 to 0.00	0.70 to 0.00	1.30 to 0.10	1.30 to 0.10	

Binocular C50 CVA	−0.04 (0.07)	0.04 (0.07)	0.13 (0.19)	0.17 (0.21)	**0.012** ^¶^
	0.00 to −0.20	0.20 to 0.00	0.50 to −0.10	0.60 to 0.00	

Binocular C10 CVA	0.11 (0.08)	0.14 (0.12)	0.28 (0.24)	0.31 (0.24)	0.059
	0.20 to 0.00	0.30 to 0.00	0.80 to 0.00	0.80 to 0.10	

^¶^Statistically significant difference between healthy subjects and glaucoma and AMD groups. Values provided are mean (standard deviation) and range. UDVA, uncorrected distance full contrast visual acuity; CDVA, corrected distance full contrast visual acuity; C50VA, 50% contrast corrected visual acuity; C10VA, 10% contrast visual acuity, AMD, age-related macular degeneration.

**Table 3:** Binocular contrast sensitivity values.

	Healthy	Glaucoma	Preperimetric glaucoma	Dry AMD	*P* (K–W)
3 cycles per degree	2.08	2.05	2.07	1.88	**0.008** ^¶^
6 cycles per degree	2.11	1.87	2.05	1.87	0.085
12 cycles per degree	1.65	1.28	1.62	1.35	**0.016** ^§^
18 cycles per degree	1.17	0.87	1.16	1.00	0.095

^¶^Controls versus dry AMD. ^§^Controls versus glaucoma.

**Table 4:** Results of the Catquest-9 questionnaire.

Questions	Healthy	Glaucoma	Preperimetric glaucoma	Dry AMD	*P*
1. Does your vision cause you difficulties?	3.45 (1.04)	3.67 (0.50)	3.44 (0.73)	3.11 (0.60)	0.241
	1–4	3-4	2–4	2–4	

2. Are you satisfied with your vision?	3.50 (0.71)	2.89 (0.33)	3.22 (0.67)	2.33 (0.50)	**0.003** ^1^
	2–4	2-3	2–4	2-3	

3. Do you have difficulties reading text?	3.64 (0.67)	3.22 (0.67)	3.33 (1.00)	3.00 (0.87)	0.202
	2–4	2–4	1–4	1–4	

4. Do you have difficulties recognising faces?	3.91 (0.302)	3.78 (0.44)	3.78 (0.44)	3.56 (0.53)	0.338
	3-4	3-4	3-4	3-4	

5. Do you have difficulties seeing prices?	3.73 (0.65)	3.44 (0.73)	3.67 (0.71)	3.11 (0.93)	0.178
	2–4	2–4	2–4	1–4	

6. Do you have difficulties walking on irregular surfaces?	3.80 (0.42)	3.89 (0.60)	3.67 (0.71)	3.44 (0.89)	0.726
	3-4	3-4	2–4	2–4	

7. Do you have difficulties doing manual work?	3.30 (0.95)	3.44 (0.72)	3.50 (1.07)	3 (0.00)	0.127
	1–4	3- 4	1–4	3	

8. Do you have difficulties watching TV?	3.36 (0.92)	3.33 (0.87)	3.67 (0.50)	3.11 (1.27)	0.756
	1–4	2–4	3-4	1–4	

9. Do you have difficulties when engaged in your hobbies?	3.55 (0.93)	3.44 (0.53)	3.56 (0.72)	3.43 (0.53)	0.674
	1–4	3-4	2–4	3-4	

^1^ *P* = 0.042 for preperimetric glaucoma versus dry AMD and *P* = 0.002 for healthy subjects versus dry AMD. For question 2, the scores awarded for the possible options are as follows: 1, very dissatisfied, 2, rather dissatisfied, 3, fairly satisfied, and 4, very satisfied. For the rest of the questions, the scores for possible answers are as follows: 1, very great difficulty, 2, great difficulty, 3, some difficulties, and 4, no difficulties. Thus, a higher score represents better patient-perceived outcomes.

## Data Availability

The dataset analysed during the current study is available from the corresponding author upon request.

## References

[1] Yeu E., Cuozzo S. (2020). Matching the patient to the intraocular lens: preoperative considerations to optimize surgical outcomes. *Ophthalmology*.

[2] Alio J. L., Plaza-Puche A. B., Férnandez-Buenaga R., Pikkel J., Maldonado M. (2017). Multifocal intraocular lenses: an overview. *Survey of Ophthalmology*.

[3] Braga-Mele R., Chang D., Dewey S. (2014). Multifocal intraocular lenses: relative indications and contraindications for implantation. *Journal of Cataract and Refractive Surgery*.

[4] Cao K., Friedman D. S., Jin S. (2019). Multifocal versus monofocal intraocular lenses for age-related cataract patients: a system review and meta-analysis based on randomized controlled trials. *Survey of Ophthalmology*.

[5] Wang S. Y., Stem M. S., Oren G., Shtein R., Lichter P. R. (2017). Patient-centered and visual quality outcomes of premium cataract surgery: a systematic review. *European Journal of Ophthalmology*.

[6] Ouchi M., Kinoshita S. (2015). Implantation of refractive multifocal intraocular lens with a surface-embedded near section for cataract eyes complicated with a coexisting ocular pathology. *Eye*.

[7] Complete publications details not given in the reference list, Query raised

[8] Gayton J. L., Mackool R. J., Ernest P. H. (2012). Implantation of multifocal intraocular lenses using a magnification strategy in cataractous eyes with age-related macular degeneration. *Journal of Cataract & Refractive Surgery*.

[9] Bach M. (1996). The Freiburg visual acuity test—automatic measurement of visual acuity. *Optometry and Vision Science*.

[10] Bach M. (2007). The Freiburg visual acuity test–variability unchanged by post-hoc re-analysis. *Graefe’s Archive for Clinical and Experimental Ophthalmology*.

[11] Grzybowski A., Kanclerz P., Tuuminen R. (2020). Multifocal intraocular lenses and retinal diseases. *Graefe’s Archive for Clinical and Experimental Ophthalmology*.

[12] Jonker S. M., Bauer N. J., Makhotkina N. Y., Berendschot T. T., van den Biggelaar F. J., Nuijts R. M. (2015). Comparison of a trifocal intraocular lens with a +3.0 D bifocal IOL: results of a prospective randomized clinical trial. *Journal of Cataract & Refractive Surgery*.

[13] Pepose J. S., Qazi M. A., Chu R., Stahl J. (2014). A prospective randomized clinical evaluation of 3 presbyopia-correcting intraocular lenses after cataract extraction. *American Journal of Ophthalmology*.

[14] Ichhpujani P., Thakur S., Spaeth G. L. (2020). Contrast sensitivity and glaucoma. *Journal of Glaucoma*.

[15] Patel S. B., Snyder M. E., Riemann C. D., Foster R. E., Sisk R. A. (2019). Short-term outcomes of combined pars plana vitrectomy for epiretinal membrane and phacoemulsification surgery with multifocal intraocular lens implantation. *Clinical Ophthalmology*.

